# Impacts of mobile health interventions on infant and young child feeding in LMICs: systematic review and meta-analysis

**DOI:** 10.1186/s12887-025-06450-6

**Published:** 2026-02-25

**Authors:** Thiwawan Thepha, Ekamjot Sangha, Debbi Marais

**Affiliations:** 1 Department of Midwifery, Faculty of Nursing, Khonkaen University, 123 Mittapap Road, Khonkaen, 40002 Thailand; 2 University of Essex, Public Health Agency of Canada, Brampton, ON Canada; 3 Warwick Medical School, University of Warwick, Coventry, CV4 7AL UK

**Keywords:** MHealth, Breastfeeding, Child health, LMIC, Systematic review

## Abstract

Infant and Young Child Feeding (IYCF) plays a critical role in child survival and development, especially in LMICs. There is evidence that mhealth interventions impact health outcomes therefore this systematic review aims to synthesize existing evidence on the impact of mHealth interventions on breastfeeding practices, complementary feeding, and nutrition-related attitudes, knowledge, and self-efficacy in LMICs. The PRISMA guidelines of reporting were followed for the searching of seven databases and screening following eligibility criteria. Of the 2393 articles screened, only 22 met the eligibility criteria, 11 were randomized trials, three were quasi-experimental studies, three were cohort studies, two were mixed methods, and two were cross-sectional surveys. The articiles were published between 2010 and 2023. Most of the studies were conducted in LMICs. Many types of mHealth intervention were reported namely, a digital job aid, voice/text messaging, telephone-counselling interventions, mobile or web-based educational applications and multi-component interventions. The meta-analysis results demonstrated that mhealth interventions significantly impact on the initial breastfeeding (BF) (OR = 1.25, 95% CI = 0.91–1.73), exclusive breastfeeding (EBF) duration at 1-month (OR = 1.55,95% CI = 1.03–2.34), 5/6 months (OR = 1.74, 95% CI = 1.34–2.29), complementary feeding (OR = 1.51,95% CI = 1.17–1.94), and minimum dietary diversity. Future research should compare effectiveness of different mHealth, Long-term effects and sustainability modalities such as integrating into health system in LMICs.

## Introduction

Infant and Young Child Feeding (IYCF) plays a critical role in ensuring child survival, growth, and development, particularly during the first two years of life—a window of opportunity with lifelong consequences [1]. Despite global health efforts, undernutrition and micronutrient deficiencies remain widespread in low- and middle-income countries (LMICs), contributing significantly to childhood morbidity and mortality [2, 3]. Suboptimal feeding practices, such as delayed initiation of breastfeeding, low exclusive breastfeeding (EBF) rates, and inadequate complementary feeding, are major contributors to malnutrition in these regions.

Early initiation of breastfeeding and EBF are foundational components of optimal IYCF. These practices are associated with reduced neonatal infections, improved immune development, and lower risk of non-communicable diseases later in life [4], However, global EBF rates remain suboptimal at just 44%, with even lower rates in many LMICs. Key barriers include limited maternal knowledge, low self-efficacy, sociocultural norms, inadequate family or community support, and poor access to accurate information [3, 5].

Complementary feeding—introducing nutrient-rich solid and semi-solid foods at around 6 months—is equally critical, yet only 29% of children worldwide meet the minimum dietary diversity required for adequate nutrition [6]. In LMICs, this challenge is often compounded by food insecurity, poor maternal education, and limited access to quality healthcare or nutrition counseling [7]. These feeding deficits contribute not only to stunting and wasting but also to developmental delays and impaired cognitive outcomes.

To address these persistent challenges, mobile health (mHealth) interventions have emerged as a promising and scalable strategy, especially in resource-constrained settings. WHO defined mHealth is the use of mobile and wireless technologies to support the achievement of health objectives [8]. The widespread penetration of mobile phones—including in rural and underserved populations, with over 96% global coverage—positions mHealth as a feasible platform to bridge gaps in healthcare delivery [9]. Through diverse modalities such as SMS, voice messaging, smartphone apps, telephone counseling, and web-based platforms, mHealth can deliver evidence-based, timely, and personalized health information to caregivers. These interventions offer the potential to improve maternal knowledge, shift attitudes, boost self-efficacy, and encourage adherence to recommended IYCF practices [10, 11]. According to the self-efficacy theory, to encourage and increase the perception or self-efficacy could lead the good behavior [12], which mHealth was applied this theory [13].

Nevertheless, the effectiveness of mHealth interventions varies by context, content design, and modality. While multicomponent and interactive platforms show promise, one-way messaging systems and non-tailored interventions have demonstrated inconsistent outcomes, often due to low engagement, literacy challenges, and lack of cultural sensitivity. Additionally, digital divides related to gender, education, and access must be carefully addressed to avoid exacerbating health inequities.

Therefore, this systematic review aims to consolidate existing evidence on mHealth's influence on IYCF in LMICs, focusing on breastfeeding practices including the initial BF, EBF duration at 1-month, 5/6 months, complementary feeding, and minimum dietary diversity via meta-analysis. The objectives are to determine the impact of mHealth interventions on the initial BF, EBF duration at 1-month, 5/6 months, complementary feeding, and minimum dietary diversity. Additionally, in LMICs, there are many differences such as regional variations, cultural barriers, gender dynamics, or health system constraints. To explore the mHealth should be concerned all these factors as well.

## Materials and methods

This review adhered to the Preferred Reporting Items for Systematic Reviews and Meta-Analysis (PRISMA) guidelines 2020 [14]. This systematic review was registered with the International Prospective Register of Systematic Reviews (PROSPERO CRD 42022318752).

### Search strategy

A comprehensive search strategy was created by selecting key terms from the major PICO elements, as outlined in Table 1. Search terms were developed by referencing previously published systematic reviews in the field of IYCF (Table 1). Finally, the search strategy was reviewed by an institutional librarian and adapted for each of the seven databases to ensure the relevancy of retrieved records. One reviewer (ES) conducted database searches in November, 2023. A total of seven database searches (Pubmed/Medline, OVID, CINAHL, Cochrane, International bibliography of social science, SCOPUS, Web of science) were completed. The time frame was between January 2010 to November 2023 (Table 2). mHealth technologies was defined as mobile and wireless devices, to improve one of the eight IYCF indicators discussed above. Typical applications of mHealth in IYCF interventions involved the use of educational text and voice messages, apps, and patient monitoring devices [10, 15].

**Table 1:**
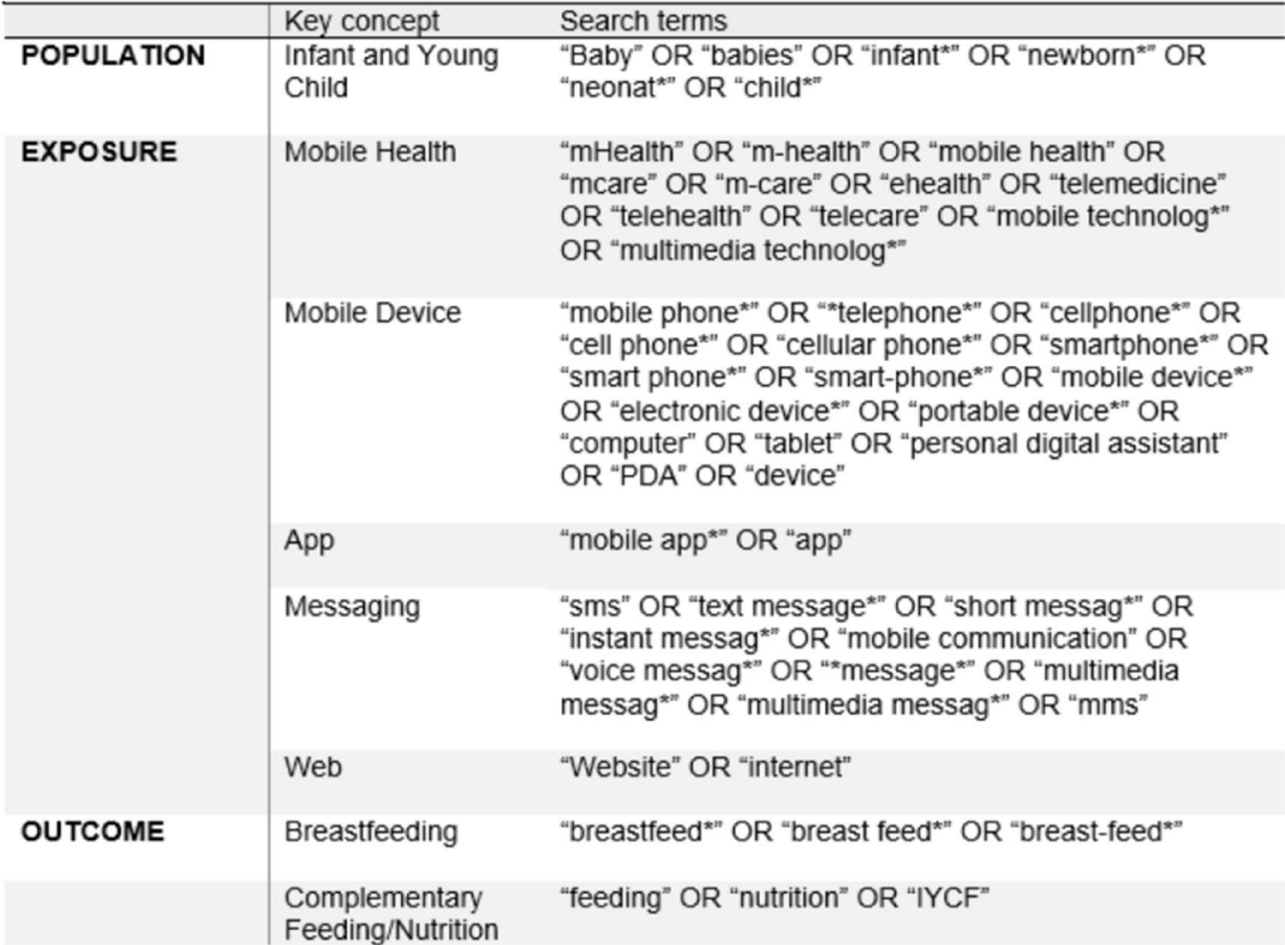
Inclusion and exclusion criteria

**Table 2:** Inclusion and exclusion criteria

	**Inclusion Criteria**	**Exclusion Criteria**
*Population*	Infants and Children from Birth to 5 Years of Age	Infants and Children with diseases that limit exclusive breastfeeding
*Setting*	Low- and Middle-income countries	High-income countries
*Intervention*	Studies involving mHealth interventions	Studies involving other health interventions, including physical mobile clinics, community health programs, and hospital-based programs
*Outcome*	1 st outcomes: Early initiation of breastfeeding, 1-month & 6-month exclusive breastfeeding, complementary feeding, minimum dietary diversity 2nd outcomes: maternal knowledge, breastfeeding self-efficacy	maternal health, pregnancy, or infant mortality
*Types of Studies/Designs*	RCTs, qualitative, and mixed-method studies that report data on IYCF outcomes	Feasibility studies, case reports, and studies focused on non-IYCF outcomes
*Type of Publication*	Papers published in peer-reviewed journals and grey literature	Policy briefs, study protocols, reports from governmental and non-governmental organizations, commentaries, systematic reviews, and oral presentations
*Language*	Studies Available in the English Language	Studies for which English translation is not available
*Publication Date*	January 2010 to November 2023	Studies Before January 2010

The inclusion criteria (Table 2) included PICO aspects, namely the key population (P) of interest was Infants and Children from Birth to 5 Years of Age. The intervention (I) was mHealth interventions, with the key comparator (C) of interest being standard care. The primary outcome (O) compared was Early initiation of breastfeeding, 1-month & 6-month exclusive breastfeeding, complementary feeding, minimum dietary diversity and the secondary outcome was maternal knowledge, breastfeeding self-efficacy. In addition, the inclusion criteria included RCTs, qualitative, and mixed-method. All papers needed to be published in a peer-reviewed journal and studies had to be conducted in countries defined as LMICs, as defined by the World Bank. Included studies had to have been published between January 2010 to November 2023. The exclusion criteria were studies conducted with Infants and Children with diseases that limit exclusive breastfeeding. Feasibility studies, case reports, and studies focused on non-IYCF outcomes were excluded. Studies for which English translation was not possible also were excluded.

### Study selection

After the completion of the database searches, results were imported into Elsevier Mendeley Desktop Reference Manager Version 1.19.8 and de-duplicated. The final list of articles was exported into RIS format and then imported into Rayyan for screening. Following this, title and abstract screening was conducted by two independent reviewers (ES and TT) using the eligibility criteria outlined such as Studies Available in the English Language (Table 2). Studies that did not meet the eligibility criteria were excluded by selecting one of the pre-determined reasons for exclusions.

Articles selected for inclusion in the title and abstract screening phase were retrieved in full and re-imported into a separate review in Rayyan. Two independent reviewers (ES, TT) reviewed the full texts of included studies and evaluated them against the eligibility criteria. Any conflicts between the two independent reviewers were resolved by a third reviewer (DM). Uncertainties regarding eligibility criteria were resolved during review team discussions, and final decisions were documented.

### Data extraction

Data extraction was conducted using an Excel data extraction template which included the following fields 1) general study information, including authors, publication year, duration, and country; 2) details of the intervention, including the type of mHealth and description of the intervention; 3) information on the study design and target population, including the number of participants, population characteristics, and sampling strategy; 4) primary outcomes and results of the study; and 5) study limitations and applications. Extraction of the data and quality assurance were conducted by both reviewer (ES, TT). Any conflicts between the two independent reviewers were resolved by a third reviewer (DM).

### Quality assessment

The Critical Appraisal Skills Programme's (CASP) Randomised Control Trial checklist was used to examine the risk of bias and data quality for all RCT studies. Accordingly, each RCT was evaluated on criteria such as randomization, blinding, treatment effect and precision. The quality of non-randomized and mixed-methods studies was examined using the Mixed Methods Appraisal Tool (MMAT). As such, quantitative non-randomized studies were evaluated on factors including the clarity of objectives, relevancy of data, sampling methods, completeness of outcome data and confounders. Similarly, mixed-methods studies were evaluated on the clarity of objectives, the relevance of data, the rationale for the design, and adequate integration and interpretation of results from each method.

### Data analysis

Data analysis was conducted by grouping studies into quantitative and qualitative categories. Then, a narrative synthesis [16] of the results of included studies was performed to determine the impacts of mHealth interventions in the field of IYCF. The data were grouped follow 1 st & 2nd outcome: the Effects on early initiation of breastfeeding, Effects on exclusive breastfeeding, Effects on Complementary Feeding and Effects on minimum dietary diversity. Quantitative findings were inputted into SPSS version 28 with a random effects model and approach to heterogeneity. The software was used to analyze heterogeneity (I2) and calculate odds ratio at the 95% confidence level. Forest plots were generated for each quantitative indicator and exported into PNG format for inclusion in the final review. Additionally, in case, studies did not provide effect estimates or measures of variability (e.g., OR, CI, *p*-values), reliability limits could not be calculated.

## Results

### Study selection

**Fig. 1:**
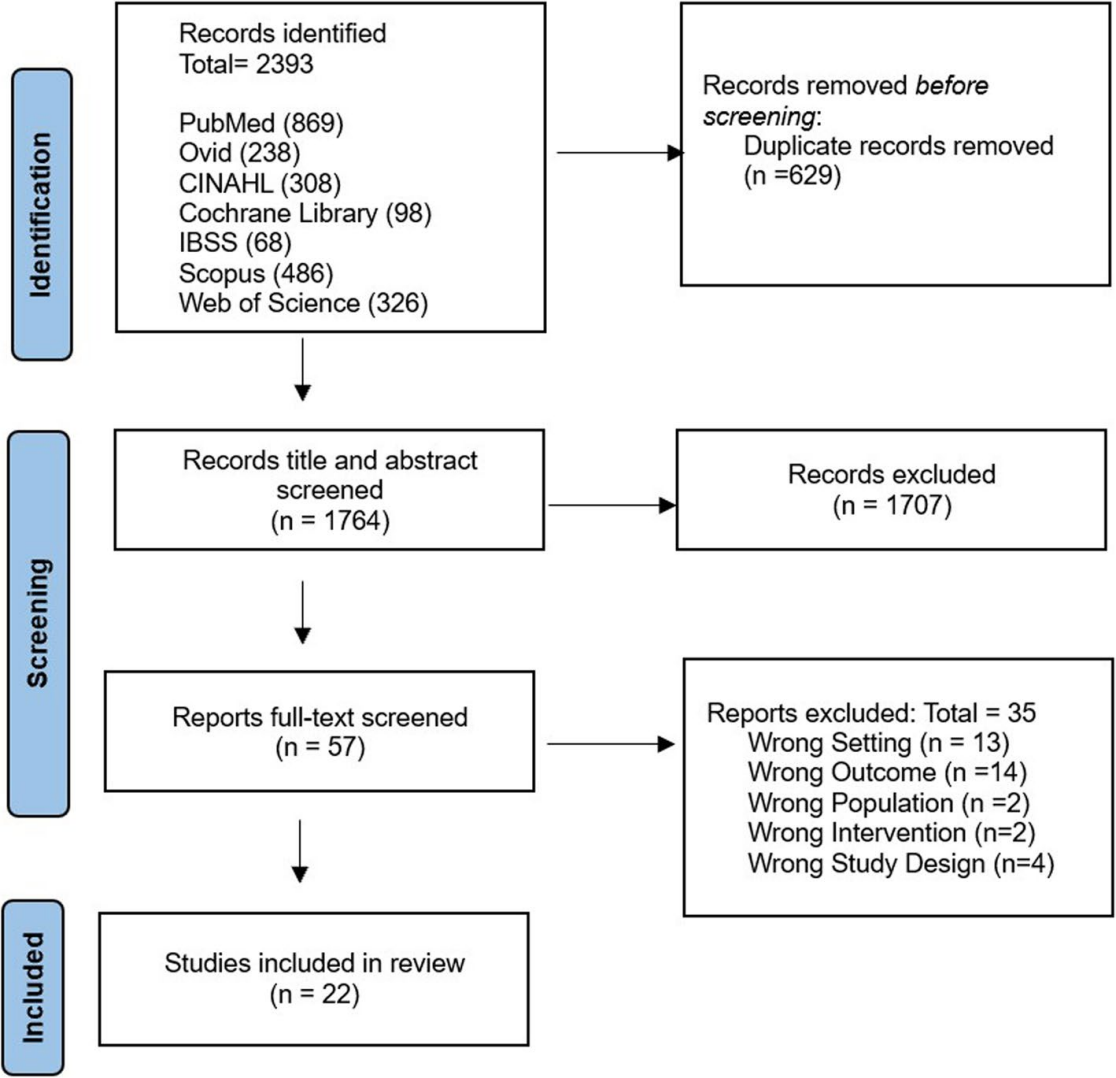
PRISMA flow

A total of 2393 records were identified from the seven databases, of which 629 were retained following de-duplication. Following title and abstract screening, 1707 records were removed. Full-text screening of the remaining 57 records resulted in the removal of 34 articles. The reasons for exclusion included: 13 studies were conducted in high-income settings, 13 studies measured non-IYCF-related outcomes, four studies were formative or feasibility assessments, two studies did not use mHealth interventions, and other two studies showed the remaining study that was conducted in HIV-positive women. The outcomes of the study selection are presented in the PRISMA diagram below (Fig. 1).

### Study characteristics

Out of 22 eligible studies, 11 were randomized trials, three were quasi-experimental studies, three were cohort studies, two were mixed methods, and two were cross-sectional surveys (Table 3). The retained studies covered 14 LMICs: India (6), Bangladesh (3), Iran (2), Nigeria (2), Brazil (1), China (1), Guatemala (1), Kenya (1), Pakistan (1), Senegal (1), South Africa (1), Vietnam (1) and Turkey (1). Studies were published between 2010—2023. For the sample size, there were 487,869 participants in total. The largest study was a RCT study in India with 476,952 participants while 13 of the studies had under 500 participants. The population targeted by most interventions were pregnant women, post-partum women and also newborns.

**Table 3:** Study characteristics

STUDY	COUNTRY	STUDY DESIGN	STUDY DURATION	SAMPLE SIZE	TYPE OF MHEALTH	MHEALTH APPLICATION	DESCRIPTION OF INTERVENTION
1. Adam et al. 2021	South Africa	RCT	November 2018 to March 2020	I = 745 C = 755	Digital Job Aid	Client Education & Behaviour Change Communication	A series of 13 short teaching videos focusing on IYCF were provided to Mentor Mothers as aids for counselling
2. Akber et al. 2019	Pakistan	Cohort	July to December 2016	135	Digital Job Aid + Text/Voice Messages	Client Education & Behaviour Change Communication Data Collection & Reporting	A mobile application was developed for registration and follow-up of pregnant and lactating women by Lady Health Workers (LHWs). In addition, bi-weekly voice and text messages were sent to recruited mothers and their families
3. Alam et al. 2017	Bangladesh	Cohort	2014	I = 210 C = 266	Voice & Text Messages	Client Education & Behaviour Change Communication	The Aponjon service consisted of voice and text messages delivered to subscribers two times per week
4. Billah et al. 2021	Bangladesh	RCT	Aug 2016 to Jan 2019	I = 855 C = 403	Digital Job Aid	Client Education & Behaviour Change Communication Data Collection & Reporting Human Resource Management	An android-based application was used to aid in scheduling and nutrition counselling by CHWs. Information collected by the CHWs was synced to a central server
5. Carmichael et al. 2019	India	RCT	Jan 2012 to Aug 2014	I = 744 C = 809	Digital Job Aid	Client Education & Behaviour Change Communication Data Collection & Reporting Human Resource Management	The information Communication Technology-Continuum of Care Service Tool (ICT-CCS) aided FLWs with registration and tracking of participants; scheduling of home visits; provision of health information via videos; guided protocols for home visits; and tracking of immunizations
6. Dodou et al. 2021	Brazil	RCT	Oct 2016 to Jul 2019	I = 120 C = 120	Telephone Counselling	Client Education & Behaviour Change Communication	Educational counselling via telephone was provided at 7-, 30-, 90- & 150-days postpartum
7. Downs et al. 2019	Senegal	Mixed Methods	Phase 1: Jun-Jul 2015 Phase 2: Apr – May 2017	Phase 1: 6 Participants Phase 2: 47 Households	Voice Messages	Client Education & Behaviour Change Communication	Voice messages targeting beliefs, attitudes and behaviours related to IYCF were delivered twice weekly
8. Flax et al. 2022	Nigeria	Cohort	May 2019 to Apr 2020	I = 600 C = 600	Text Messages & Counselling and Support Groups via Whatsapp	Client Education & Behaviour Change Communication	A multi-component intervention was delivered to participants, including printed educational materials, mobile phone messages, mass media messaging and a WhatsApp support group
9. Jerin et al. 2020	Bangladesh	Quasi-Experimental Study	Apr – Dec 2010	I = 164 C = 129	Telephone Counselling	Client Education & Behaviour Change Communication	Mobile phone counselling was provided to participants every 15 days until six months postpartum
10. Modie et al. 2019	India	RCT	Feb 2016 to Jan 2017	I = 2762 C = 2606	Digital Job Aid	Client Education & Behaviour Change Communication Data Collection & Reporting Electronic Decision Support Human Resource Management	The ImTeCHO application comprises various mobile technology-based job aids to facilitate tracking, scheduling health services, screening, counselling, decision support, real-time monitoring, and supervision of FLWs
11. Murthy et al. 2019	India	Pseudo- RCT	January 2015 to December 2017	I = 1516 C = 500	Voice Messages	Client Education & Behaviour Change Communication	The mMitra package delivered 145 voice messages from 6-weeks of pregnancy until 1-year postpartum
12. Ogaji et al. 2020	Nigeria	RCT	March to December 2017	I = 75 C = 75	Telephone Calls	Client Education & Behaviour Change Communication	Telephone counselling was provided to participants at 7- and 14-days postpartum, followed by monthly calls until the sixth month
13. Prieto et al. 2017	Guatemala	Mixed Methods	2014–2015	Phase 1–100 Phase 2–44	Text Messages	Client Education & Behaviour Change Communication	One-way and two-way text messages were sent to participants twice a week
14. Sari & Altay 2020	Turkey	RCT	2018	I = 44 C = 44	Web-Based Education	Client Education & Behaviour Change Communication	Participants were enrolled in a web-based educational program focusing on topics such as infant growth & development and breastfeeding
15. Seyyedi et al. 2020	Iran	RCT	March to December 2018	I = 110 C = 110	Mobile Phone Application	Client Education & Behaviour Change Communication	An educational smartphone application was tested with information on child nutrition, complementary feeding, and maternal health
16. Seyyedi et al. 2021	Iran	RCT	January to December 2019	I = 51 C = 51	Mobile Phone Application	Client Education & Behaviour Change Communication	An educational application was tested with information on the importance of breastfeeding, behavioural methods, complementary feeding and EBF, pumping and manual expression, managing common breast-related and breastfeeding problems, breastfeeding tips in particular situations, and answers to common queries
17. Short et al. 2021	India	Quasi-Experimental Study	2019 to 2020	I = 110 C = 112	Digital Job Aid	Client Education & Behaviour Change Communication	A mHealth tool with multi-media content was used to support peer counselling on infant and young child feeding topics
18. Unger et al. 2018	Kenya	RCT	2014	Group 1–99 Group 2–99 Control – 100	Text Messages	Client Education & Behaviour Change Communication	One-way and two-way text messages were sent to participants on topics such as antenatal care, pregnancy complications, infant health and EBF
19. Ward et alL. 2020	India	Quantitative Non-Randomized	2014–2017	Exposed – 5815 Unexposed – 12,881	Digital Job Aid	Client Education & Behaviour Change Communication	Pre-recorded audio health messages were used to aid FLWs
20. Ward et al. 2021	India	Cross-Sectional Surveys	2014–2015	Exposed – 2608	Digital Job Aid	Client Education & Behaviour Change Communication	FLWs used mobile phone-based audio recordings covering content about RMNCH behaviours
21. Wu et al. 2020	China	RCT	May 2019 – April 2020	I = 170 C = 174	Mobile Phone Application + Text Messages	Client Education & Behaviour Change Communication	A WeChat optimal feeding module provided information on IYCF and had an online forum for questions. In addition, educational messages were sent to the intervention group three times each week
22. Doan et al. 2022	Vietnam	Triple-blinded RCT	2020–2022	I = 634 C = 632	Mobile Phone Application	Client Education & Behaviour Change	mHealth provided three messages per week and linked with the information in the application's library content to improve breastfeeding practices

There are many types of mHealth intervention types. Out of 22 included studies, seven involved using a digital job aid; five used voice or text messaging; three used telephone-counselling interventions; four used mobile or web-based educational applications; and three used multi-component interventions.

### Results of critical appraisals

Following the critical appraisal of all included studies using the CASP RCT Checklist and MMAT checklists for quantitative non-randomized and mixed-methods studies, 20 of 21 studies were deemed acceptable for inclusion in data analysis.

Two mixed methods were assessed using the MMAT checklist for mixed-methods studies; the results are summarized in Table 4 below. Both studies met five out of seven criteria and did not have an adequate discussion of inconsistencies between the qualitative and quantitative aspects of the studies. For the study by Downs et al. [17], the quantitative portion was limited by small size, lack of a control group, and a short follow-up period. The pilot quantitative study conducted by Prieto et al. (2017) was limited by non-randomized sampling, high loss-to-follow-up and small sample size.

**Table 4:** Results of critical appraisal using the mmat – mixed methods

P = Meets Criteria O = Does Not Meet Criteria? = Insufficient information							
Citation	Clear Objectives	Relevant Data	Rationale for Design	Integration of Results	Adequate Interpretation of Results	Inconsistencies Addressed	Adherence to Quality Criteria of Each Method
7.Downs et al. 2019	P	P	P	P	P	?	O
13. Prieto et al., 2017	P	P	P	P	P	O	O

The results of the critical appraisal of five studies using the MMAT quantitative non-randomized checklist are presented in Table 5 below. The quasi-experimental study by Akber et al. [18] was excluded due to non-randomized sampling, failure to account for confounding factors, and non-comprehensive reporting of the study results. Studies by Alam et al. [19] and Flax et al. [20] did not have samples representative of LMICs; for the former, the participants were recruited from a list of paid service subscribers, while for the latter, participants were exclusively recruited from private health facilities.

**Table 5:** Results of critical appraisal using the mmat – quantitative non-randomized

P = Meets Criteria O = Does Not Meet Criteria? = Insufficient information							
Citation	Clear Objectives	Relevant Data	Representative Sample	Appropriate Measurements	Complete Outcome Data	Confounders Accounted	Intervention/Exposure Administered as Intended
2. Akber et al. 2019	P	O	O	O	?	?	P
3. Alam et al., 2017	P	P	O	P	P	P	?
8. Flax et al. 2022	P	P	O	P	P	P	?
19. Ward et al. 2020	P	P	P	P	P	P	?
20. Ward et al. 2021	P	P	P	P	P	P	?

Fifteen RCTs were appraised using the CASP RCT checklist. It was noted that most studies lacked information on cost-effectiveness and harms, preventing an assessment of the benefit-bisk. The common methodological limitations were observed, including inadequate or unclear blinding, insufficient reporting of baseline comparability, and possible inconsistencies in treatment delivery between groups. Five studies did not provide confidence intervals, while *p*-values were not reported for two studies. A lack of generalizability was noted for many studies due to factors such as recruitment from specialized centres, short follow-up periods, high median age, a high proportion of post-secondary education, and a small sample size (Table 6).

**Table 6:** Results of critical appraisal using the casp rct checklist

P = Meets Criteria O = Does Not Meet Criteria? = Insufficient information											
Citation	Focused	Randomisation	Drop-Out	Blinding	Baseline Similar	Equal Treatment	Treatment Effect	Precise	Benefit/Risk	Generalisable	Value
1.Adam et al. 2021	P	P	P	?	P	P	P	P	?	P	O
4.Billah et al. 2021	P	P	P	O	P	O	P	P	?	P	O
5.Carmichael et al. 2019	P	P	O	?	P	P	P	O	?	P	?
6.Dodou et al. 2021	P	P	P	P	?	P	P	O	P	O	P
9. Jerin et al. 2020	P	O	P	O	P	P	P	O	?	O	P
10.Modi et al. 2019	P	P	P	O	P	P	P	P	P	O	P
11.Murthy et al. 2019	P	?	P	O	P	P	P	P	?	?	O
12.Ogaji et al. 2020	P	P	P	?	P	P	P	P	O	O	O
14.Sari & Altay 2020	P	P	P	O	P	P	P	O	?	O	P
15.Seyyedi et al. 2020	P	P	P	P	P	P	P	P	?	O	P
16. Seyyedi et al. 2021	P	P	P	?	P	P	P	P	P	O	P
17.Short et al. 2021	P	O	P	O	O	P	P	P	?	?	P
18.Unger et al. 2018	P	P	P	O	P	P	P	P	?	O	P
21.Wu et al. 2020	P	P	P	O	P	P	P	O	?	O	P
22.Doan et al. 2022	P	P	P	P	P	P	P	O	P	P	P

#### 1^st^ outcomes

##### Effects on early initiation of breastfeeding

**Fig. 2:**
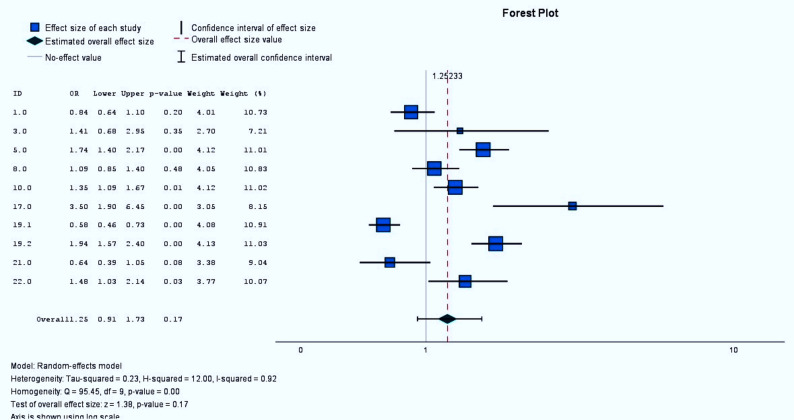
Effects of mhealth interventions on early initiation of breastfeeding

Nine studies reported the impacts of mHealth interventions on rates of early breastfeeding initiation; the results are summarized in Fig. 2. According to meta-analysis, the overall, the estimate for the pooled effect is a odds ratio of 1.25. The 95% CI was 0.91–1.73, therefore this result is not statistically significant. It is mean the digital health intervention not significantly affected to early initiation of breastfeeding at the 95% confidence level. However, a high degree of heterogeneity between the studies was found (I2 = 92%) (Fig. 2).

##### Effects on exclusive breastfeeding

**Fig. 3:**
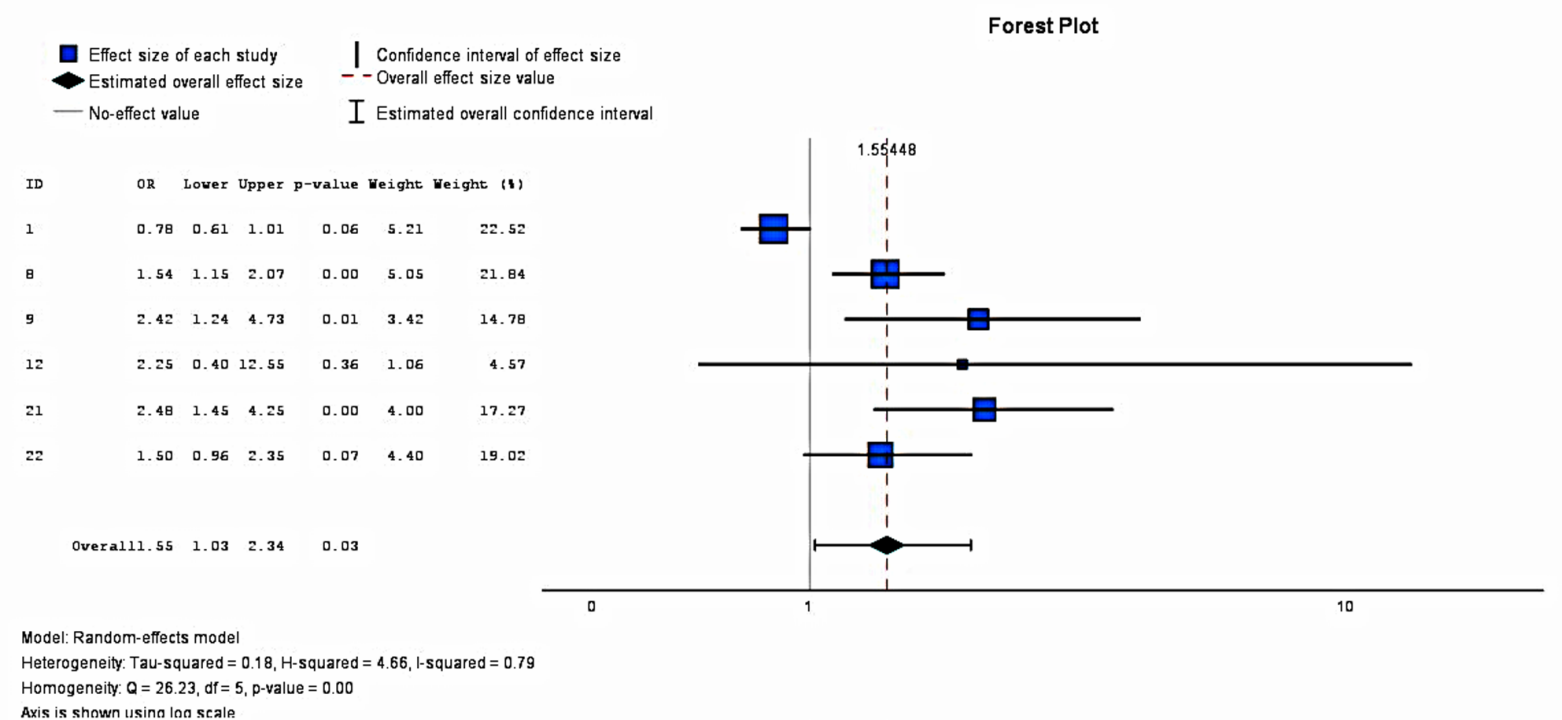
Impact of mHealth Interventions on EBF Rates at 1-Month

Concerning rates of EBF at one month, data from five studies were extracted, and the results are presented in Fig. 3 below. As a forest plot, a heterogeneity analysis found a high heterogeneity level between studies (I^2^ = 79%). The pooled effect was estimated as a odds ratio 1.55 (95% CI = 1.03–2.34). This value was statistically significant at the 95% confidence level. Moreover, statistically significant impacts on EBF rates were observed in only one study: Wu et al. (2020) [*p* < 0.01]. Adam et al. [13] reported lower rates of EBF in the intervention group than in the control group at one month; however, the results were not statistically significant [p = 0.09]. Although favourable results were reported in studies by Jerin et al. (2020) and Ogaji et al. (2021), no *p*-values were reported.

**Fig. 4:**
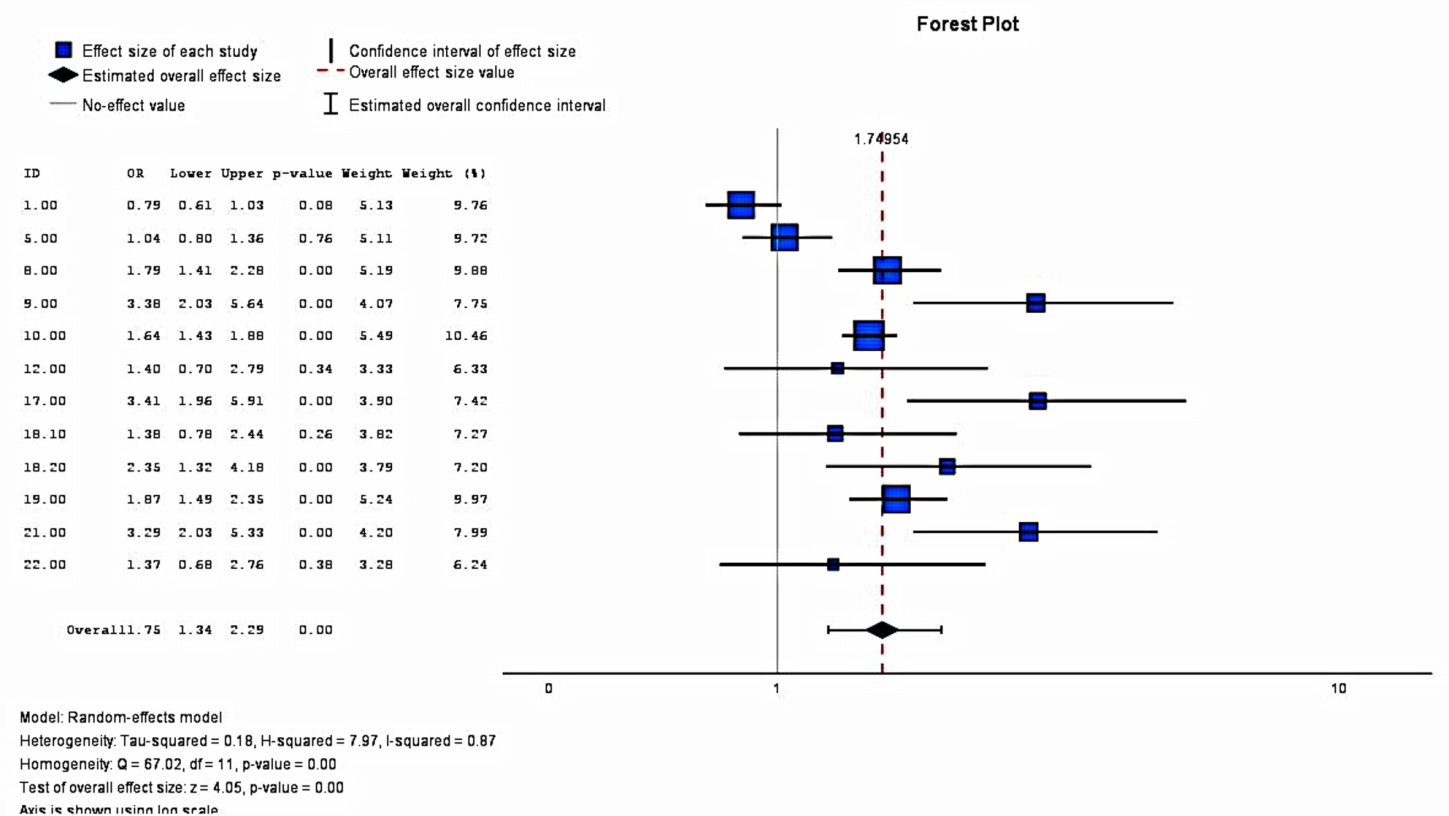
Impact of mHealth Interventions on EBF Rates at 5 to 6 Months

At the 5 to 6-month time point, data from ten studies were extracted and presented in Fig. 4. The pooled effect was estimated as a odds ratio (1.74, 95% CI = 1.34–2.29); this finding was significant at the 95% confidence level. A heterogeneity analysis found a high heterogeneity level between studies (I^2^ = 87%). Additionally, nine studies reported positive results on rates of EBF; of these nine studies, findings from six were statistically significant. In the mixed-methods study by Adam et al. [13], the intervention group had lower breastfeeding rates than the control group (*p* = 0.152). After accounting for confounding factors, the risk ratio at the 5-month time point was calculated to be 0.82 (0.67 to 1.02, *p* = 0.074).

##### Effects on complementary feeding

**Fig. 5:**
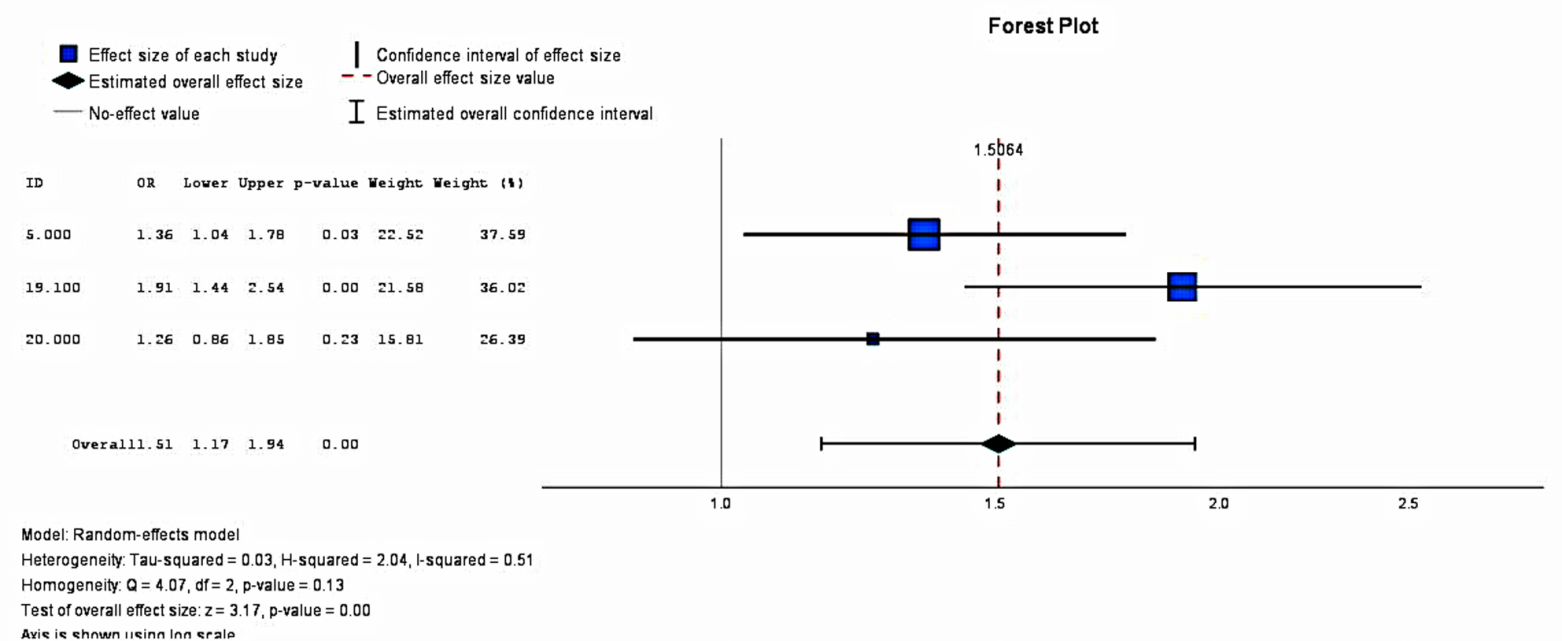
Impact of mHealth interventions on timely introduction of solid foods

A total of three studies reported on the timely introduction of solid foods; the extracted data is presented in Fig. 5 below. All three studies reported higher rates of timely introduction of solid foods in intervention groups than in control groups. In addition, the meta-analysis found medium heterogeneity between the three included studies (I^2 =^ 0.51%) and the odds ratio was 1.51 (95% CI = 1.17–1.94). This result was significant at the 95% confidence level.

##### Effects on minimum dietary diversity

**Fig. 6:**
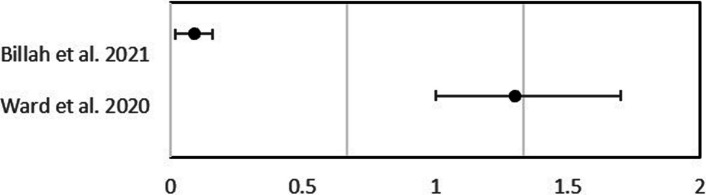
Impact of mHealth interventions on dietary diversity

This systematic review identified two studies that reported on mHealth interventions' impacts on dietary diversity scores. As depicted in Table 7, studies by Ward et al. (2020) reported positive effects on dietary diversity scores. However, Billah et al. [21] indicated a non-meaningful effect on dietary diversity scores. In both studies, the *P*-values were not reported for either study; therefore, the statistical significance of the results is unknown. Figure 6 presents the odds ratios associated with the Dietary Diversity Scores.

**Table 7:** Minimum dietary diversity

study	Dietary Diversity Score	Control Group	Odds ratio	*P*-Value
4. Billah et al. 2021	2.39 [± 1.39]	2.30 [± 1.38]	0.09 (0.02–0.16)	Not Reported
19.Ward et al. 2020	1.84 [± 0.16]	1.42 [± 0.05]	1.3 (1.0–1.7)	Not Reported

#### 2^nd^ outcomes

##### Impacts on maternal knowledge

Several studies measured the impact of mHealth interventions on maternal knowledge and intentions related to IYCF topics. In the mixed-methods study by Adam et al. [13], the impacts of the mobile video intervention delivered by CHWs were measured using a 15-point knowledge assessment at two-time points. A modest increase in maternal knowledge was observed within the intervention group at the one-month (OR = 1.04, 95% CI = 1.01–1.07, *p* = 0.012) and five-month timepoints (OR = 1.03, 95% CI 1.00–1.06, *p* = 0.084) which is not statistically significant.

In the RCT by Murthy et al. (2019), the intervention group had a statistically significant increased odds of knowing that babies should be given solid foods by six months of age (OR = 1.89, 95%CI = 1.371–2.605, *p* < 0.01). However, the intervention did not have statistically significant impacts on knowledge indicators related to early initiation of breastfeeding (*p* = 0.065) or exclusive breastfeeding (*p* = 0.396).

Prieto et al. (2017) investigated the effects of a text-messaging intervention on exclusive breastfeeding knowledge and found this intervention was effective in increasing EBF knowledge in participants. Wu et al. (2020) used the social media app WeChat to deliver informational messaging on various IYCF topics, such as the duration of breastfeeding, the introduction of complementary foods and early initiation of breastfeeding. This intervention did not significantly impact any of the four knowledge outcomes.

Two mHealth interventions in Bihar, India, significantly impacted mothers' knowledge of breastfeeding and infant nutrition practices. First, Ward et al. [22] investigated the effects of two job aids: Mobile Kunji and Dr.Anita voice messages. At the 3-month follow-up point, knowledge scores for mothers in the intervention job were significantly higher than in the control group (20.23 vs. 16.02, *p* < 0.001). A similar intervention investigating the impacts of the GupShupPotli audio recording found that mothers in the intervention group were 1.6 times more likely to have knowledge of complementary feeding [23].

##### Effects on breastfeeding self-efficacy

Three studies investigated the impacts of mHealth interventions on self-efficacy. The RCT conducted by Dodou et al. [24] found that telephone-based counselling and motivational interviewing improved self-efficacy at all time points (60, 120, and 180 days). In addition, the intervention group was reported to have higher breastfeeding self-efficacy than the control groups at all time points, but only the results at the 60-day time point were statistically significant. Sari & Altay [25]investigated the impacts of a web-based educational intervention and found that mean parental self-efficacy scores were significantly higher in the intervention group compared to the control (p < 0.05). Finally, Seyyedi et al. [26] found that an educational smartphone application produced a 26.45-point difference between the intervention and control groups (*p* < 0.001). The baseline score of intervention group and control group was 119.08 and 125.10. The final score of intervention group and control group was 145.92 and 125.50.

For mix-method research, the results shown that m-health reduced the burden on the mentor mothers to perform 100% of the health counseling verbally, the mentor mothers perceived a “freeing up of time.” In addition, the mentor mothers felt that the videos increased the consistency of messaging across participants and improved general interest in infant feeding. In another research show that participants reported enjoying receiving the voice messages and found it helpful.

Several limitations of mHealth have been identified. The intervention tended to be more effective among women with higher levels of education, greater income, and those living in urban areas, whereas women from poorer, less-educated, and rural backgrounds were less likely to benefit. Cultural norms such as early bathing of newborns due to beliefs about birth-related impurity persisted despite exposure to mHealth messages, highlighting the limited ability of digital interventions to shift deeply ingrained practices, particularly in extremely poor communities. While mHealth was associated with short-term improvements in dietary diversity, such as increased consumption of eggs and flesh foods, these effects were not maintained beyond 12 to 15 months of age. In addition, the cost-effectiveness of had not been explored.

## Discussion

This systematic review of mobile health interventions on infant and young child feeding to LMIC identified several important points. The use of digital job aids has emerged as a valuable tool for improving IYCF practices, particularly breastfeeding, in (LMICs). These digital tools can enhance healthcare workers' performance, improve the delivery of IYCF counselling, and improve confidence among workers [22, 27, 28].

When designing an intervention involving a digital job aid in a low-resource setting, several factors should be considered to ensure the intervention is feasible and effective. Firstly, researchers should have an understanding of the local context, including cultural norms and health practices [29]. Next, the intervention should be compatible with the existing technological infrastructure and be functional in areas with limited electricity or internet access [17]. When designing the intervention, the needs of the end-users should be understood, including their language needs and cultural sensitivities [17]. The cost-effectiveness of the intervention should be analyzed to ensure sustainability [10]. Lastly, there should be plans in place for training, monitoring and evaluation and long-term sustainability of the intervention [30]. Overall, the evidence suggests that digital job aids can be an effective tool in promoting optimal breastfeeding and IYCF practices. However, the effectiveness of these interventions may vary depending on the context and specific digital tools used. Further research is needed to optimize the development, implementation, and scaling of interventions using digital job aids.

This review’s meta-analysis on the impact of mHealth interventions on 1-month and 5/6-month time point data found a statistically significant impact on EBF rates. Moreover, the results from the studies suggest that mHealth interventions may effectively promote the timely introduction of solid foods and potentially improve dietary diversity scores in LMICs. However, researchers must be aware of the multifaceted and interconnected factors that impact child-feeding decisions when designing interventions. In addition, tailored interventions to address the unique challenges faced by families in LMICs, such as food insecurity, will help improve indicators related to IYCF, including dietary diversity and meal frequency.

In addition, mHealth interventions can potentially enhance maternal knowledge of IYCF practices, with variable success depending on the specific topic and format of the intervention. This is consistent with existing literature, which indicates that mHealth interventions can positively impact maternal knowledge and behaviour related to IYCF practices in LMICs. Further research is needed to identify the most effective mHealth strategies for each setting and to determine best practices for developing and delivering educational information to impact maternal knowledge and intentions. However, women from poorer, less-educated, and rural backgrounds are less likely to benefit. This may be due to limited access to technology, economic constraints (such as not owning a phone or using low-quality devices), low digital literacy, and unreliable connectivity. To enhance the effectiveness of mHealth interventions, these factors should be carefully considered in program design and implementation.

This systematic review analyzed three studies examining the effects of mHealth interventions on self-efficacy, with positive results in each case. Self-efficacy is a critical construct in the field of IYCF due to its impact on breastfeeding intentions and rates. Research has shown that mothers with higher self-efficacy are more likely to initiate breastfeeding, exclusively breastfeed for six months, and successfully continue breastfeeding for extended periods (Zubaran et al., 2010). In addition, mothers in this category are better equipped to manage breastfeeding difficulties, ensuring a more successful breastfeeding experience (Otsuka et al., 2014). Interventions that focus on increasing maternal self-efficacy, such as skill-building, problem-solving, social support, and goal-setting, have positively impacted breastfeeding rates (McQueen et al., 2011). Therefore, targeting maternal self-efficacy through various educational and counselling interventions aids in promoting optimal breastfeeding practices and improving overall infant health.

There are the strengths and limitation. A primary strength of this systematic review was its methodological rigor in following PRISMA guidelines. In addition, this study was registered to PROSPERO to ensure compliance with standards. This rigour ensures the reproducibility of the study and reduces the risk of bias. Secondly, through the use of a robust and comprehensive search strategy, this review ensures that data from multiple sources was captured. The search strategy was vetted by librarian to increase the likelihood of identifying relevant studies and reducing the risk of missing important sources of information. A further strength of this study was the use of critical appraisal tools and quality checks to ensure the validity of the data and minimize bias. At each step of the review, oversight was provided by an independent reviewer to maintain accuracy and adherence to PRISMA guidelines. There are several limitations, such as the lack of risk of bias assessment, absence of sensitivity or subgroup analyses, missing funnel plots or small-study effect evaluations, and the omission of confidence intervals or *p*-values. Additionally, the generalizability of the findings is limited. These shortcomings may reduce the reliability and effectiveness of the results and should be addressed in future research. Future research could focus on examining the effectiveness of different mHealth intervention delivery methods, assessing cost-effectiveness to inform long-term impact, and evaluating the acceptability of interventions among target users to ensure sustainability.

## Conclusion

This systematic review indicates that mobile health interventions have a beneficial impact on infant and young child feeding in LMICs. Future research should compare effectiveness of different mHealth, Long-term effects and sustainability modalities such as integrating into health system in LMICs.

## Data Availability

No datasets were generated or analysed during the current study.
